# Speaking Through Silence: The Lonelification at the Core of Domestic Abuse

**DOI:** 10.1177/10778012251323268

**Published:** 2025-03-12

**Authors:** Sara Skoog Waller, Ulla Forinder

**Affiliations:** 1 Department of Occupational Health Science and Psychology, University of Gävle, Gävle, Sweden; 2 Department of Social Work, Criminology and Public Health Sciences, 3485 University of Gävle, Gävle, Sweden

**Keywords:** domestic violence and abuse, coercive control, loneliness, identity, gaslighting

## Abstract

This article is based on narratives from 20 women who have experienced domestic violence and abuse (DVA). Based on in-depth interviews, we explored their lived experiences of the mechanisms and meanings of loneliness in the context of DVA. The women experienced social and existential loneliness, not as passive consequences of victimization, but through active isolating and *lonely-making* tactics inflicted on them by the abusers, as well as through responses from personal and professional networks and institutions. We present the concept *lonelification* to offer a framework for the understanding of lonely-making as a core aspect of DVA, which targets women's sense of self, reality, and connectedness.

My friends, family, relatives … One by one they had to go. But it was always my decision to make. He indoctrinated me that they were so dirty and bad - one was a whore and the other one was a whore. But I was always the one who had to make the decision, and he always said in the end that “Yes, but you must decide for yourself.” He wanted to make it look like it was me, even though he was the one who decided. And little by little everyone disappeared, and eventually, I had no contact with anyone.

The woman quoted above participated in the interview study on which this article is based, where we explored experiences of loneliness among victim-survivors of domestic violence and abuse (DVA). The quote captures loneliness, not as a passive consequence, but as an active process orchestrated by the abuser through tactics that disrupt the woman's sense of self and reality. The focus of this article is to decode the processes, mechanisms, and meanings of loneliness based on victim-survivors’ lived experiences of DVA.

## Introduction

Feeling socially connected with others in time and space is essential for the fulfillment of basic needs and for one's sense of self (Guisinger & Blatt, 1994; Wickramaratne et al., 2022). Access to social support is of particular importance for abilities to cope with hardships and to recover from trauma (Kaniasty, 2012; Saltzman et al., 2018), while the absence of social support has been found to exacerbate negative consequences of trauma (Wang et al., 2018). Experiences of negative social support are not only related to more severe posttraumatic stress symptoms but have also been identified as a stronger predictor of trauma severity than the event or situation that caused the trauma in the first place (Zoellner et al., 1999).

In DVA and its aftermath, social isolation, loneliness, and lack of social support appear to be central aspects (Engnes et al., 2012; Rokach, 2006, 2007). DVA is characterized by systematic control and restriction of the victim's freedom over time (Dobash & Dobash, 2004; Stark, 2007; Stark & Hester, 2019). The abuse can be described as complex patterns of actions, performed by the abuser to maintain *coercive control* over the victim, in relation to a patriarchal order of dominance over women (Dobash & Dobash, 2004; Donovan & Barnes, 2021; Stark, 2007). To uphold such control, the abuser uses different tactics to stay present through time and space—monitoring, microregulating, and punishing any deviation from the order—with a constant gaze into the victim's everyday life, referred to as *omnipresence* (Johnson, 2008; MacMillan, 2022; Spearman et al., 2023; Stark, 2012). Social isolation has been highlighted as a central mechanism in DVA and the notion that abusers seek to isolate their victims has become popular through the power-and-control wheel (Pence & Paymar, 1993). It is also recognized that, along with socially isolating tactics, abusers use manipulative tactics to distort the victim's sense of reality, a tactic often referred to as *gaslighting* (Sweet, 2019). It has been suggested that the entrapment in the unreal world staged by the abuser, restricts the victim's possibilities to fully comprehend or share their perceptions of reality in general, and of the abuse in particular (Williamson, 2010). The unreality is orchestrated by the abuser's efforts to distort the victim's perceptions, memory, and *sense of self*, targeting anything from insignificant details in daily life to more major events in the victim's personal life stories and personal characteristics (Sweet, 2019). Specifically, the abuser may distort the course of events regarding his own abusive acts and the victim's reactions, making it look as if the victim was responsible for his abuse and that her reactions are pathological and exaggerated, that is, the woman is crazy or irrational (Ferraro, 2006; Stark, 2007; Sweet, 2019). Unreality or gaslighting could also be understood as a state of existential loneliness in terms of the unbridgeable gap between the victim's sense of self and the abusers’ claims of who she is, which may target the underpinnings of the victim's identity (Drummond, 2021). Existential loneliness might also be found in the gap between the victim's actual experience of reality and the reality as pictured by the abuser, and sometimes by institutional and structural responses (Sweet, 2019). In the present study, we draw on concepts and ideas about *coercive control*, *omnipresence*, *unreality, gaslighting, and identity* to understand the processes of loneliness in DVA.

Although conceptions about the relationships between DVA and social isolation are widespread, findings from empirical studies are inconsistent (Coohey, 2007; Erez et al., 2009). Previous studies have found that women subjected to DVA have social networks as big as women who are not (Tan et al., 1995). Yet, other studies report that women who are subjected to DVA perceive their received social support as weaker (Carlson et al., 2002). While the social isolation of victims of DVA has been attributed to socially isolating tactics that are adopted by abusers (Heron et al., 2022), less attention has been paid to social isolation related to responses from personal and professional networks outside of intimate relationships. However, Bellotti et al. (2021) suggest that social isolation is a result of the complex dynamics of responses from the support networks as well as the women themselves, emphasizing the responses from family, friends, and professionals. Furthermore, several studies performed during the COVID-19 pandemic found that social isolation and decreased movements outside the home were related to increased DVA victimization in the population (Agüero, 2021; Boxall et al., 2020; Leslie & Wilson, 2020; Ravindran & Shah, 2023). Thus, empirical data do point to the conclusion that social isolation and DVA are related. However, the relationship between DVA and loneliness is less studied. While social isolation represents an objective state of absence of social contacts and interactions, loneliness represents the personal experience of lacking close ties or a sense of community with others, or of feeling socially and existentially cut off and abandoned (Goodman & Epstein, 2020; Sand & Strang, 2006). The focus of this article is the personal experience of feeling lonely emotionally or psychologically—experiences that are associated with suffering such as feelings of meaninglessness and lack of purpose in life (Stillman et al., 2009; Zeligman et al., 2019), as well as serious risks to physical and mental health (Holt-Lunstad, 2017; Holt-Lunstad et al., 2010). Different forms of loneliness have been described in the literature and we will focus on two separate forms of loneliness: social loneliness which is related to the lack of intimate social relationships, and existential loneliness which constitutes experiences of being separated from the world and others in the world irrespective of having close relationships and acquaintances or not (Bolmsjö et al., 2019). Existential loneliness is derived from the experience of lacking a genuine and empathetic listener who understands one's feelings and thoughts, and from unfulfilled needs of a true communicative relationship that sheds light on our true selves, as well as the stories of who we are and what we have been through (Bolmsjö et al., 2019).

Importantly, processes of loneliness can be expected to depend not only on the abuser's controlling tactics, but also on responses from social networks, such as friends and family, and professional networks, such as social workers and healthcare professionals. These networks may be crucial as to how and *if* the violence is recognized and understood. In other words, response networks may influence the possibilities for victims to share their experiences of DVA and to receive help and support (Hydén et al., 2016). Moreover, responses to violence communicate to the abuser and the victim whether or not the violence is accepted or even recognized as violence (Hydén et al., 2016). Thus, the continuation and escalation of violence, as well as the possibilities for victims to be heard and understood, depends on responses from professionals and social networks (Hydén, 1994, 2015; Hydén et al., 2016). This article aims to deepen the understanding and knowledge of the processes by which women exposed to DVA become lonely, addressing not only the abuse in the relationship but also its embeddedness in social and institutional contexts and responses.

### Purpose

The purpose of this article was to explore lived experiences of social and existential loneliness among women exposed to DVA by a previous partner. More specifically, the purpose was to conceptualize the mechanisms of loneliness in the context of DVA as well as the meaning of loneliness in women's everyday lives.

## Method

### Participants

Twenty Swedish-speaking women participated in the study. The inclusion criteria for participation in the study were women who were 18 years or older who had been exposed to DVA by a previous intimate partner. Demographics and details about the life situations of the participant group are displayed in Table 1. Participants were recruited via information about the study on women's shelter pages on social media (Facebook and Instagram) and those who wished to participate could choose to contact the researchers.

**Table 1.** Overview of Demographics and Life Situations of the Participants in the Study.

*N*	Age span	Years passed since separation	Years in relationship with the abuser	Number of women who had children with the abuser	Country of birth (number of women from each area)	Number of women who had received protection interventions (e.g., sheltered accommodation, protected identity and/or restraining order)
20 women	22–75 years (*M* = 40)	1–34 years (*M* = 7)	1–24 years (*M* = 7.5)	10	Sweden: 16 Other European country: 3 Non-European country: 1	10

### Data Collection

In-depth interviews were conducted from Spring 2020 until Spring 2021. Two women participated in three interviews, 11 women participated in two interviews, and seven women participated in one interview. The interviews lasted 1–3 h, except for one follow-up interview which lasted for 30 min. The interviews were teller-focused (Hydén, 2014) with a narrative approach (Mishler, 1986). The interviews started with one or two open-ended questions from the interview guide followed by the women's stories, with supportive and clarifying questions. The interviews started with the question, “Would you like to start by telling me a bit about yourself?.” Often, this question was enough to prompt the women to share their experiences on the topic for the interview. If the woman did not spontaneously get onto the relationship with the abuser, the following question was asked: “Would you like to tell me about your relationship with the abuser and what your life looked like when you met?” The interview guide included four areas: (1) The abuser and the relationship; (2–3) personal and professional networks in different phases (pre- and postseparation) and their responses; (4) experiences of social support and isolation or loneliness. The interviews were recorded and transcribed.

### Data Analysis

The interview data was analyzed through thematic analyses (Braun & Clarke, 2006) to identify patterns and recurring themes in the women's narratives (Table 2). The first step in the analysis was to listen to and read the transcripts several times. The second step was to identify meaning units in the interviews and to develop a system for coding these meaning units into more condensed meaning units, or codes. The same text chunk could be coded with several overlapping codes. The third step was to create subthemes into which the codes could be divided. The fourth step was to develop main themes representing exclusive sections of the subthemes. The identified themes are presented in the findings section.

**Table 2.** Examples of the Procedure in Conducting the Data Analysis.

Meaning unit	Code	Subtheme	Theme	Level
He would put recording devices in my car and at home and follow me to work and sure enough he would call and ask “What are you doing there?”	Surveillance	Omnipresence and unipresence	Disruption of existence	Interpersonal
	Spatial restrictions			
	Temporal restrictions			
They just said “Oh that's terrible.” Nothing more. Although I did tell them that I was afraid that he was going to kill me. I was devastated and they just sent me home, to him.	Minimization of violence	Neutralization of abuse	Disruption of reality	Institutional
	Silence and silencing			
	Responsibility without power			

The present study has been evaluated and approved by the Swedish Ethical Review Authority. For many of the women who participated in the study, there could be certain risks related to sharing details about their life stories or about the abuser, that could be tied to their own, their children's, or the abuser's identity. For that reason, several ethical considerations have been made. When we present quotes in the *findings and analysis* section, we do so without fictitious names or other labels. This won’t allow the reader to follow the separate stories of individual women through the text, but the risks for identification of the women are smaller. The risks regarding the participant's personal integrity have also been managed through careful procedures in the handling of personal information in accordance with the GDPR principles (European Parliament Council of the European Union, 2016). Participants will remain anonymous for others than the researcher conducting the interviews and confidentiality will be maintained. The participants were informed about the purpose and proceedings of the study through an information letter. Informed consent was obtained from the participants, and they had the possibilities to ask the researchers questions before, during, and after participation.

## Findings and Analysis

The purpose of this article was to study lived experiences of social and existential loneliness among women who had been subjected to DVA by a previous partner, and to conceptualize the processes, mechanisms, and meanings of loneliness in the context of DVA. In this section, we present results based on the thematic analyses of the women's narratives about experiences of loneliness in the context of DVA.

**Figure 1.**
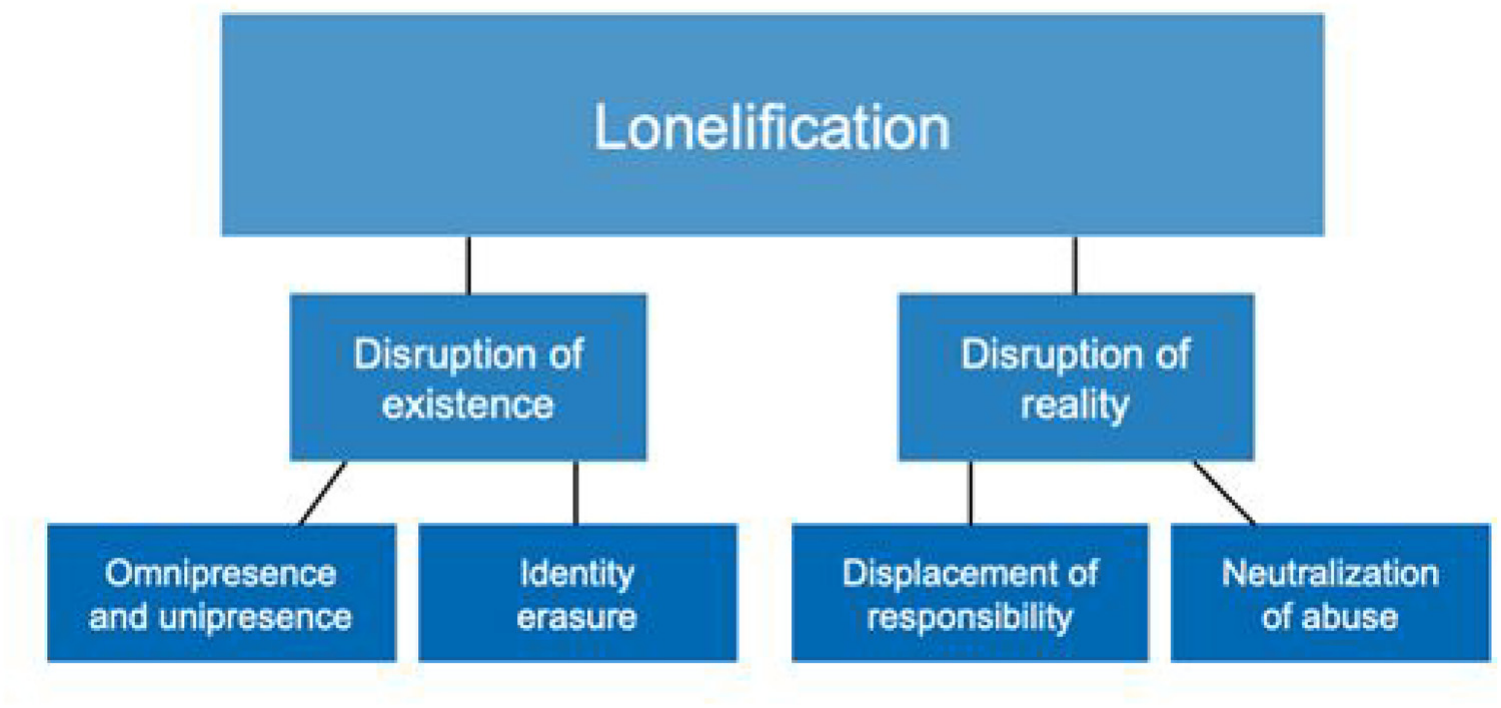
A model illustrating the concept of lonelification, its meanings (two themes) and mechanisms (four subthemes).

The women interviewed in the study described social isolation and loneliness as core aspects and overarching aims of the abusers’ violence and control. In most cases, the abusers had actively aimed to disconnect the women from their social contacts and contexts. In our analysis, we identified four subthemes representing categories of tactics that made the women socially and existentially lonely: *Omnipresence and unipresence, Identity erasure, Displacement of responsibility,* and *Neutralization of abuse*. These four subthemes formed two main themes representing the meaning that lonely-making had in the women's lives: *Disruption of existence* and *Disruption of reality*. In turn, these two subthemes make up the overarching concept *Lonelification* that we present in this article (illustrated in Figure 1).

### Disruption of Existence

The main theme *Disruption of existence* captures experiences shared by women in this study, of being targeted with controlling tactics aimed at undermining their personhood and self-identification and limiting their autonomy in time and space in order to regulate their relationships, interactions, and possibilities to connect with others.

#### Omnipresence and Unipresence

The subtheme *Omnipresence and unipresence* is one of two subthemes that underpin the main theme *Disruption of existence*. While the concept omnipresence is described in the introduction, we propose the term *unipresence* to illustrate that abusers not only claim to be omnipresent, but also exclusively present in the woman's mind. Unlike omnipresence, where the perpetrator establishes control from the outside in, unipresence means that the perpetrator tries to control the woman from the inside out. Indeed, several women described the abuser as being omnipresent, through limitations of their temporal and spatial autonomy, upheld by different types of surveillance. Yet, more often than not, the omnipresence appeared to aim at regulation of the women's possibilities to interact with others. As one woman put it:

I had no job and no financial security, and he had control over our money. He had the car keys. Well, everything. We lived in the countryside, and the only way to get around was by car, then he set up a system to hide car keys. He could unscrew spark plugs from the car and things like that. He could let me go shopping, but it couldn’t take too long because then he called and checked.

On the surface, the controlling tactics could be interpreted as jealousy directed against other men. However, the social limitations were general rather than specific. For instance, the abuser might forbid or punish the woman's contacts with colleagues, clients, or friends: “He was very angry when I started to work. In his opinion, me going to work meant that I was unfaithful to him. He thought I was loose because I was working, because I talked to other people and socialized.”

The abusers used controlling tactics to establish social limitations, not despite the fact that they weren’t physically present at all times, but rather because they weren’t physically present all the time. For many of the women, the safest way to avoid abuse was by balancing their own freedom against its risks and costs, as described by one of the women: “It made me see friends less often. It wasn’t nice to be distrusted when I was only seeing a girlfriend and he was sure I would meet a guy. And then he would check me physically when I came home. He would put his hands up inside me, to make sure that I wouldn’t smell anything but myself.”

The women's social connectedness with others than the abuser, gradually decreased, and in many cases, the women lost access to entire social spheres such as networks of friends, family members, colleagues, and schoolmates. This meant that the women, after some time in the relationship, often found themselves caught in a state of *fluid entrapment* where they felt that they had nothing and no one without the abuser—something that was also described as an active strategy adopted by the abusers:

He also didn't like me talking on the phone with someone else. And it could be my family, or my best friend. It could be my grandmother and he wouldn’t like it. He wanted to get his message across that I didn’t have anyone else. ‘Without me you are all alone.’ I only had him, and if I didn’t have him I would be all alone and have nothing.

The abusers made use of different possibilities to control and interfere with the women's interactions in real time as well as in retrospect. Control and interference were often technology-facilitated. Communication and information technologies, including digital media and surveillance devices such as cameras installed in the home, were used to keep constant contact as well as to monitor the women. There were also examples of low-tech approaches such as placing objects in certain positions before leaving the home and checking the positions later to tell what the woman had been up to. These tactics enabled the abuser to directly monitor, intervene with, and restrict the woman's social life: “It was a constant terror of text messages when I was at work, when I was in meetings. He would put recording devices in my car and at home and follow me to work and sure enough he would call and ask “What are you doing there?” And he got into my phone and into my iPad and stuff.”

Unlike omnipresence, unipresence was typically reflected in the abuser's requirements of totalitarian access to attention and affection at all moments. Activities that implied that the woman could have interacted with others, were confronted by the abuser as threats that called for actions to re-establish unipresence. Activities that offered the women more freedom, if only for a moment, such as starting a new project at work, having coffee or taking a walk with a friend or speaking to a neighbor, could thus escalate the abuse: “But anything that threatened his control over me triggered the violence. And the greater the threat to his control, the more serious the violence. And more life-threatening.”

One woman described an event of physical and sexual violence that lasted for several hours while her 2-year-old child was in bed in the next room. The abuser had just come home from a week-long business trip and started interrogating the woman who she had met while he was away, alternating between questioning her and what can best be described as physical torture. There were several similar stories of how the abuser escalated his abuse in response to loss of control.

Women who had children with the abuser or from previous relationships were often forced to make impossible priorities between mothering and risk reduction. The mother–child relationship, the children's immediate needs of attention, responsiveness and care, and the women's willingness to fulfill those needs appeared to pose threats to the abusers’ unipresence, and abuse was often triggered in situations where the children needed attention, care, and comfort. The women described having to balance motherhood against the risks of “*making things worse”* for the children.

He demanded hundred percent attention all the time so I couldn’t put the children to bed and read a book to the children. He got upset if I wanted to put the kids to bed and he didn’t get the attention, and … I wasn’t functioning as a mother. I never had time for the kids. And I often feel like, you know, I could leave. Even though it still affects me, I could actually leave. But the children cannot. They are still with him.

#### Identity erasure

Another subtheme under the main theme *Disruption of existence* was *Identity erasure*, which represents tactics that isolate and undermine the woman's identity and sense of self. The women described that little by little they were stripped of their personhood and possibilities to express their individuality, competence, or personal preferences of, for example, clothing, interior design, music, or foods. They experienced how the abuser tried to isolate and disconnect their identities from their personal histories, social and relational contexts, and the places in which they were grounded. This meant that the women could have to dissociate their own perceptions of their authentic selves in order to keep abuse from escalating. One woman described how the abuse started with a critique of her physical appearance and escalated to the point where she did not know if she would survive: “There was nearly lethal violence and threats that he would kill me. It went from him controlling what I would wear to him almost taking my life.”

In many cases, abusers would attack the women's identities related to their professional roles or their roles in personal life. For example, the abuser could demonstrate the relative unimportance of the woman's parenthood through sexually abusive acts deliberately performed in the children's presence, such as rape, touching the woman's private parts in front of the children or talking about sexual acts he would force her to endure. These tactics can be understood as attacks on the mother–child relationship, but also as tactics to disrupt the woman's identity as a mother: “He was the most important person all the time. Not me, nor the kids. It was always him and his needs that were most important. So, he couldn't wait for me to put the children to bed. Even when the children cried and I wanted to comfort them, it was still his need for sex that came first.”

Several women had repeatedly endured violence so severe that they were close to dying. One woman described the threat of lethal violence as the ultimate expression of the abuser's power over her existence: “He decided if I lived or died.” In several cases, the abusers had made explicit descriptions of what they would do to the women's dead bodies, which added to the sense of depersonalization and helplessness. As one woman put it:

He yelled right into my face. And in the middle of this frantic screaming, he kind of became completely calm, and then he leaned forward to my face very cold and still and said, “I'm going to cut your body up and put it in plastic bags.” I froze to ice because I felt that he could really do it.

The women described how their existence was rendered meaningless independently of the abuser. Their previous life stories were often targeted by the abusers, who in more or less subtle ways made it clear that anything that resembled the women's previous relationships or life contexts was unspeakable and had to be eliminated. In some cases, any material objects or signs of a previous life story needed to be eliminated:

He sold everything in my house, new things, old things that I’d saved for my daughter. He sold, threw out, or gave away everything. Barbie dolls, photos, the sofa, cutlery, my new shoes, my expensive pans. But I never even saw the money. He made me change my phone number. Everything had to go. It was up to him to make me a new woman and he was the only one who could make a woman out of me. And without him I wouldn’t be a woman. I wouldn’t be anything.

### Disruption of Reality

The main theme *Disruption of reality* consists of two subthemes: *Displacement of responsibility* and *Neutralization of abuse*, capturing experiences that the women had of being subjected to tactics and responses aimed at distorting their perceptions of reality, specifically targeting their rationality and understanding of the abuse.

#### Displacement of responsibility

*Displacement of responsibility* is one of the two subthemes under the main theme *Disruption of reality*. It represents tactics and responses aimed to disrupt the woman's sense of reality by isolating her psychologically with her experiences of being subjected to violence and abuse. The abusers would minimize violent events, neutralize abuse by claiming that the women were responsible for making it happen, by denying that the abuse ever took place or claiming that she was emotionally unstable. Thus, the women endured constant incongruency between the outer staged reality that they were trapped in and their actual perceptions and evaluations of their lived experiences. In the reality created by the abuser, the woman was held responsible for causing the abuse and he could even claim to be the actual victim. Many of the women experienced a dissonance between their inner intentions and needs, and their own manifest actions. To avoid and resist abuse, and to protect their true selves and realities, they needed to act as if the abusers’ images of the reality were their own: “No, I didn’t do many activities alone with the children. He wouldn’t let me. He constantly told me I had to love him most and that the children came in second place. So, I became very emotionally cold toward the children because I wasn’t allowed to show them that I loved them. But in my mind I always thought differently.”

The women learned in detail how to adapt their behavior to reduce the risks of violence and abuse. Eventually, their everyday lives resembled something akin to a walk in a minefield, where the slightest misstep could have disastrous consequences. Yet, regardless of their efforts, the abusers could always find, or make up, signs of deceit or reasons for being provoked. In the end, the women experienced that the abuse was unescapable—a realization drawn from endless loops of shifting between sparks of hope, anxiously avoiding any missteps and falling through the depths of helplessness again: “Because you are used to being beaten, and you are used to protecting the children. And you’re used to seeing what mood he's in. You learn. You can sense that he's annoyed, that I need to watch out. But sometimes it doesn’t matter what you do. He just wants to exercise his power.” Importantly, many women stated that deep inside they always knew that the abuse was wrong, and that the abuser was responsible for his acts. Yet those insights remained isolated and unarticulated as they had no place in the unreality staged by the abuser. To resist abuse, the women had to adapt their sense of reality to the surreal one created by the abuser, keeping their own perceptions of reality dissociated and protected.

The women frequently expressed how impossible it felt to tell anyone about the abuse, and a few of them even said that they would probably rather have died than tell anyone. However, the women took those risks as they expected to be met with validation and support and that they would be offered help or protection. Yet many of them were met by silence, minimization, and denial when they told professionals about the abuse. One woman described that early in the relationship with the abuser, shortly after their first child was born, she tried to find a way out of her situation by telling their family counselors, provided by the municipality (the same authority that runs the social services), about the violence, even though the abuser was present during the session: “It was so unreal. I was sitting there telling the therapists about the violence and they wanted me to try to take his perspective and put the responsibility on me. Like it was just any relational problem.” The counselors instructed her to consider what she could do to make him feel less in need to control and dominate her. She described how unexpected and surreal the counselor's advice seemed to her, and how it made her fall into what she referred to as a black hole. She also explained that the experiences of being held accountable for the abuse by those she expected would offer help, convinced her that there was no way out. After the session at the family counselors, she suffered severe depression and later she would have no memories of the following year—the first year of her baby's life. After that, she stayed with the abuser for more than 10 years and had more children with him. During this period of time, he subjected her and the children to severe violence and abuse as well as escalating control.

#### Neutralization of abuse

*Neutralization of abuse* is the second of two subthemes under the main theme *Disruption of reality*. In contrast to the other subthemes, this one mainly represents responses from professionals, rather than tactics adopted by the abuser. In many cases, the women themselves had taken the initiative to tell professionals about the abuse, expecting a strong response directed toward protection and support, but felt that they had been met with disregard and minimization. The women explained that they experienced a much deeper dimension of loneliness when violence and abuse was silenced and normalized by professionals, as compared to when it had been silenced and normalized by the abuser. The reluctance, in the healthcare system, to take notice of the abuse that the women disclosed, by verbally telling and by showing their injuries, had far-reaching consequences for several of the women.

I told them that “I can't have a child with this person because he beats me.” I told two different nurses on two different occasions, but it's not even in my record. It's not written anywhere. They just said “Oh that's terrible.” Nothing more. Although I did tell them that I was afraid that he was going to kill me. I was devastated and they just sent me home, to him. And then in the investigation, the police asked for the medical record. Because I said that I had told the health care, but it was nowhere to be found.

Another woman had driven alone with spinal injuries to the emergency room after she had been severely beaten by her husband. He had slashed her car tyres, probably to stop her from going, but she managed to make the drive late at night. She described an endless loneliness related to what she experienced as a disproportionately mild and avoidant response from the hospital staff.

You feel so alone. And so late at night. On those occasions you wish that they could take you to some safe accommodation or something. I told them the truth, that he had pushed me down the stairs. And I remember there was someone who asked if I thought he would show up there. And I just replied that “I don't know where he is, and he will probably not come to the emergency room.” And then it was just like, they just let me go. No references to where I could get help. Nothing.

One woman described how she hoped to receive help from the healthcare center to make a police report and to find protection for herself and her unborn child. The nurse did not seem to even consider the danger the woman and her unborn child lived under or how the abuse would proceed, but rather made a medical assessment of the clinical severity of her physical injuries.

I was so scared. At night I didn't dare to sleep. What if he strangles me and kills me. And my teenage children said, “Mom, go to the doctor at the health care center.” And so, I went there. To the nurse, and told her the truth, and I was completely bruised around the eyes, bruises on my body and wounds on my face. “Well, the injuries aren't that serious,” she said. “Really?” But I thought they were serious and how would I know what had happened to the baby?

The rejection of the woman's story and attempt to get access to help meant that her only escape plan was blocked and she stayed with the abuser. The abuse continued, with escalating control after the baby was born. She left the abuser permanently after a couple of years, following an occasion of prolonged and nearly lethal abuse in the presence of her child.

Several women described how they had been met with silence and silencing as well as acceptance and minimization of abuse, when they reached out for help from professionals. They experienced that the abuser's view of the abuse as being something negligible and justifiable had been confirmed through these responses. They described it as a process of isolation where they were left all alone with their perceptions of the abuse as wrongful and serious. One woman described how responses from healthcare professionals made her question her own perceptions of being subjected to DVA: “I should have received so much more help. But then I felt that since they don't react, maybe it wasn't so bad, and if it had been serious, I would have gotten help.”

A recurring pattern was that healthcare services interpreted consequences of abuse as psychiatric conditions with unspecific causes, that abuse was not even considered as a possible cause for the conditions, and that the women never got a chance to tell the healthcare services about the abuse.

I had minimal support, and the support I had was not helpful. Going to the child and youth psychiatry services was really bad because my boyfriend was present at all the sessions. And it's in the psychiatry journal that my parents sit in private conversations and talk about how they’re worried that he won’t be able to cope with me because I’m so difficult. This put me in a position where I was very, very lonely and felt that there was no help available. There was no one around to help me.

Even in cases where it was established that women needed help for the psychological consequences of being subjected to DVA, professionals could ignore the topic during therapy sessions. One woman described that she was offered 11 sessions with a psychiatric counselor, upon seeking help for her traumas, but that the abuse was ignored during the sessions: “We didn't talk about the violence at all. We talked about everything else. Everyday things and how I was doing with my panic attacks, and “is it hard to ride the bus and how is it going at work?.”

Another woman explained how responses of silence, upon her telling professionals about the abuse, made her question her own perceptions of the abuse even taking place: “For me, it has not only been a silence. That silence has also always meant questioning the validity of the experience. For me, it has been the case that if I am not allowed to talk about it, did it even happen?.”

Women in this study described that professional's interpretations of their reactions to abuse, such as fear, anxiety, and despair, corresponded to attributions made by the abusers, of the women's reactions as “crazy” and exaggerated. Rather than allowing for interpretations of the reactions as adequate consequences of abuse, several of the women had experienced that professional's responses seemed to ignore the abuse and focused on the women's reactions, symptoms or injuries as the problems, or psychiatric diagnoses, to be treated, further neutralizing and silencing their narratives and testimonies about abuse.

## Discussion

The insights reported in this article provide an in-depth account of the lived experiences of loneliness among women exposed to DVA. These insights may extend the understanding of how DVA make women lonely, through processes that are entangled with structural and institutional responses. Our findings have implications for policy and practice, as well as for the research field, by offering a conceptual framework that depicts loneliness as a multidimensional, multilayered, and ongoing process in the context of DVA. Our findings also have implications for women who are or who have been exposed DVA and for their personal networks. Furthermore, the insights from this article have applications for stakeholders and for professionals who meet women exposed to DVA, not least by highlighting the importance of social support and validating professional responses to DVA. Our main result is that the women became socially and existentially lonely not as a passive result of victimization, but through active isolating and “*lonely-making”* processes operating across several dimensions and levels. We suggest a concept that we define as *lonelification* to illustrate the parallel processes by which abusers make women lonely through violent and controlling acts targeting their social contexts and identities, thus disrupting and un-grounding their sense of self. In this article, we also propose the term *unipresence,* as an extension of the concept *omnipresence*. Through this extension, we aim to illustrate that the abuser not only makes himself constantly present in order to monitor and control the woman's social interactions (Johnson, 2008; MacMillan, 2022; Stark, 2012). He also demands the impossible—proof that he is exclusively present in the woman's mind and able to control her interactions from the inside out.

As described in previous research, loneliness played a prominent role in women's everyday lives (Engnes et al., 2012; Rokach, 2006, 2007). Yet, we found that the abusers’ control and entrapment (Stark, 2012; Stark & Hester, 2019) were centered around making the women isolated and lonely, socially as well as existentially, independently of time and space.

The women knew that it was possible they were being monitored by the abuser at any time (MacMillan, 2022). In many cases, the abuser had used different types of *technology-facilitated* surveillance or *tech abuse* tactics to reinforce control and keep a constant gaze into the women's everyday lives (Havard & Lefevre, 2020; MacMillan, 2022). Importantly, the control appeared to revolve around establishing and maintaining a state of unipresence, where the abuser was constantly and exclusively present in the woman's physical and psychological reality. Furthermore, we found that the abuser would target the foundations of the woman's *identity* and personhood, in ways that disrupted her possibilities to claim her own existence and place in the world, independently of the abuser. The tactics adopted by the abusers included attempts to eliminate the woman's individuality, personal history, or at least her expressions of it, her social connectedness with others as well as her material possessions. These tactics had a striking similarity to those described by Goffman (1961) through the term *mortification*, applying to the attacks on identity that prisoners in *total institutions* such as concentration camps and prisons endure. The correspondence between mortification in total institutions and the attacks on self and identity in DVA has recently been pointed out by Neale (2023). Goffman as well as Neale highlight the relationship between loss of contact with the outside world and *identity erasure*—a connection that was also clear in the present study. The women described how their sense of who they were had become isolated, distorted, and dissociated. Through the abuse, the women had been deprived possibilities to express personhood, identity, and belonging, which can be understood as a state where the self is left disconnected, ungrounded, and isolated from meaning (Knez, 2014; Neale, 2023; Wheeler & Bechler, 2021).

At some point however, all women in this study had reached out to tell someone about the abuse, hoping to be heard and validated. Yet telling others required them to find the voice that the abuser had tried so hard silence, and they needed to use that voice to articulate experiences that could feel impossible to talk about due to trauma (Van der Kolk et al., 2005) and in fear of escalated abuse as a consequence of their disclosure. However, telling others was not only an act of finding a voice, but often even more so an act of speaking through silence—silences streaming from several barriers. To be able to disclose abuse the woman needed to break through the barrier founded on stigmatization of women who speak about their own exposure to abuse and by doing so identifies a man as a perpetrator (Mattsson, 2013; Reidy et al., 2023). Several women talked about how shame and attempts to avoid stigmatization had hindered them from seeking help (Overstreet & Quinn, 2013) and made them withdraw from social contexts where their victimization risked being made visible. Drawing on Hydén's research (Bellotti et al., 2021; Hydén, 1994, 2015; Hydén et al., 2016), the present study challenges the widespread image that DVA essentially is a hidden problem and that victims of DVA are irrational for not disclosing abuse. Instead, alongside their victimization, the women in this study provide a counter-image of *agency* and *resistance* (Wade, 1997). The women did what they could to avoid abuse and control by proactively adapting and diminishing their expressions of individuality and identity as well as their social interactions and contacts with family, friends, and colleagues. One way to understand these responses can be in terms of internalization of the abuser's demands and perspective. However, in many of the cases such explanations did not apply as the women explained that they had been aware all along that the abuse was wrongful. Another way to understand the women's adaption to the abuser's more or less explicit demands and microregulations in everyday life (Stark, 2007) is in terms of resistance within the small space for action that the women had (Kjellberg, 2023; Wade, 1997)—what might look like submission can be understood as agency. Furthermore, expectations of stigmatization (Overstreet & Quinn, 2013) did not appear to be irrational because when the women spoke up about the abuse, the responses from others were often characterized by silence, minimization, and shifts of blame and responsibility from the abuser onto the woman, thus denying the victimhood that the women were trying to make visible. Such responses have been described in previous research (Bruno, 2018; Eriksson, 2003; Laing, 2017; Pratt-Eriksson et al., 2014). However, the present study illustrates how responses of silence and silencing, particularly from professionals, were harmful and potentially dangerous since they made the women isolated with their notions of abuse and alone with the abuse itself. The responses of silence and minimization from professionals fed into the abuser's unreality in which the abuse had been normalized, denied, and blamed on the women (Laing, 2017; Stark, 2007)—a process that can be understood as gaslighting on an institutional level where representatives from social support systems participate in describing the victim as weak and oversensitive, and the abuser as acting without intention to hurt (Johnson et al., 2021; Torino et al., 2018). Importantly, Sweet (2019) highlights that to perform gaslighting, abusers draw on gendered structural and institutional inequalities and widespread tendencies that associate femininity with irrationality. Several women in the present study described how the impact of abuse on their health and well-being was interpreted as psychiatric diagnoses by medical and psychiatric services—an interpretation that reinforced and confirmed the abuser's view of the woman as irrational, emotionally unstable, crazy, and mutually responsible for the abuse. A few of the women explained how they themselves started to doubt their own sanity because of professionals’ neutralization of abuse, which they encountered when they themselves tried to reach out for help. The unreality could manifest itself at a linguistic level in the rephrasing of abuse, defining it as fights, conflicts, or relational issues, thus making the abuse invisible and unspeakable. Even severe physical and sexual violence could be rephrased as conflicts or relational problems by professionals. These findings emphasize the importance of knowledge and awareness across professional fields about specific patterns of gender-based violence in intimate relationships. Furthermore, our results show that lonelifying professional responses had far-reaching consequences for women in this study, including being left alone in traumatic situations, starting to question one's own experiences of the abuse, being denied access to resources and seeing no other option but staying with the abuser. Our results also demonstrate how existential loneliness arose from the lack of an authentic communicative relationship to bridge the gap between the surreality that the women tried to escape, and the world outside that they aimed to be a part of (Bolmsjö et al., 2019). The women tried to bridge the gap by seeking professional help and by disclosing abuse. Yet, in many cases, they found themselves alone on that bridge, speaking into silence. It should be emphasized that the passive responses of professionals put the women in dangerous positions, as any resistance against the abuser could be associated with risks of increased violence and control (Stark, 2007). Professionals who did not respond to the women's accounts of abuse with validation and empathy, or at all, missed important opportunities to address the abuse and to make the woman feel safe about sharing her experiences. Thereby they also missed possibilities to obtain more information about the situation and to offer support, for example, by documenting the abuse or by helping the woman to access adequate agencies and resources. By responding with minimization, avoidance, or silence, professionals became part of the lonelifying processes that we have described in this article, sometimes mirroring the abuser's perspective (Keeling & Fisher, 2015). In that sense, passive responses from professionals could maintain and reinforce the abuser's entrapment of the woman, socially and existentially. Corresponding with previous research in the field of trauma and social support (Wang et al., 2018; Zoellner et al., 1999), women in this study explained that the passive responses, the lack of social support, and the feelings of loneliness it caused, were more traumatic than the abuse itself.

Although Sweden is often described as leading in gender equality, women's exposure to domestic violence is consistently prevalent, with higher reported prevalence rates than most other European countries (FRA, EIGE, Eurostat, 2024), and victims still face numerous challenges related to help-seeking, lack of protection, inadequate responses from authorities and professionals, and restricted possibilities to enjoy safety, freedom, and quality of life in the aftermath of DVA (Swedish Gender Equality Authority, 2023; The National Board of Health and Welfare, 2024). Several reforms regarding policy and practice have been implemented in Sweden in recent years with the aim of improving protection and support for women and children exposed to DVA (Albért et al., 2019; The National Board of Health and Welfare, 2023). For example, reforms have been made in areas such as criminal law, family law, in responsibilities of social authorities, and in guidelines for increased identification of DVA in health care. A main part of the lonelifying responses described in this article took place in structural and professional contexts involving challenges that are still present in the Swedish context—many of them taking place in current conditions. Thus, the women's experiences can be taken to be relevant and topical to the present day.

Our study has implications for professionals from several areas, such as health care, social work, and the justice system. While it is recognized that isolation is a key tactic adopted by abusers (Bellotti et al., 2021; Pence & Paymar, 1993), this study contributes to a deeper and broader understanding of the central role of loneliness and lonely-making in DVA not only in terms of social isolation and social loneliness, but also in terms of disruption of the victim's sense of self and personhood as well as her sense of reality. Professional, institutional, and state responses and interventions can at best have the function of bridging the social and existential disruptions caused by DVA, and at worst perpetuate or mirror it. Once a woman exposed to DVA reaches out to seek help, it may have been preceded by escalated control and long periods of abuse, and the possibilities for her to explicitly articulate her situation may be limited for several reasons (Heron & Eisma, 2021; Tarzia et al., 2020). Moreover, the time frame for help-seeking may be small and occur at a critical juncture with risks of escalated abuse in the absence of adequate professional responses (Anderson & Saunders, 2003; Campbell et al., 2017; Spencer & Stith, 2020). Therefore, it is essential that professionals in several fields have the awareness, resources, and practices needed to identify, acknowledge, and respond to DVA with empathy and validation (Heron & Eisma, 2021)—matters that are dependent on how stakeholders and policy makers address, understand, and prioritize DVA and the needs of victim-survivors. Our study points out that a focus on surface-level behaviors and symptoms might leave little space for the narratives and lived experiences of abuse that are awaiting to be articulated and attributed meaning.

When we asked the women about their reasons for participating in this study, many of them explained that they wanted to bear witness to what they had been through. They wanted to give testimony about the social and existential loneliness at the core of the abuse—lonelifying processes that were reflected on an institutional level in the responses from authorities such as social services and the justice system, as well as in responses from the healthcare system. This article might give the women an opportunity to speak through the silence they experienced in response to some of their previous disclosures on DVA, and we hope that their stories will be heard.
